# Blowing the lid off! Bottle-directed, extractive foraging strategies in synurbic bonnet macaques *Macaca radiata* in southern India

**DOI:** 10.3389/fpsyg.2022.973566

**Published:** 2022-12-09

**Authors:** Arijit Pal, Santanu Mahato, Jean-Baptiste Leca, Anindya Sinha

**Affiliations:** ^1^Animal Behaviour and Cognition Programme, National Institute of Advanced Studies, Bangalore, India; ^2^Biopsychology Laboratory and Institution of Excellence, University of Mysore, Mysore, India; ^3^Department of Psychology, University of Lethbridge, Lethbridge, AB, Canada; ^4^Centre for Neuroscience, Indian Institute of Science, Bangalore, India; ^5^College of Humanities, Exeter University, Exeter, United Kingdom

**Keywords:** object manipulation, complex foraging strategies, behavioral innovation, inter-individual variability, human-macaque interface, anthropogenic environments

## Abstract

Nonhuman individuals and groups, living in anthropogenic landscapes, often adopt adaptive foraging strategies, mediated by their day-to-day interactions with humans and their artefacts. Exploring such novel behavioral manifestations, especially in the Anthropocene, offers us insights into behavioral innovations and their transmission in such rapidly changing ecologies. In this study, employing field experiments, we investigated an example of human-induced, extractive foraging behavior – the extraction of liquid contents from plastic bottles – in a synurbic bonnet macaque *Macaca radiata* population. The main aims of the study were to examine the distribution, diversity, inter-individual variability and intra-individual flexibility of bottle-directed manipulative behaviors, and to explore the social and environmental factors driving this behavioral practice. We video-recorded the manipulation of partially filled plastic bottles and the extraction of liquid across four groups of bonnet macaques in southern India. Two socio-demographic factors – age class and group membership – and one environmental factor – food provisioning – were identified as major determinants of inter-individual variation in the performance of sophisticated manipulative techniques and in bottle-opening success. Our results also suggest that age-related physical maturation, experiential trial-and-error learning, and possibly social learning contributed to the acquisition of foraging competence in this task. These findings illuminate the mechanisms underlying inter-individual behavioral variability and intra-individual behavioral flexibility amongst free-ranging individuals of a cercopithecine primate species, traditionally known for its ecological adaptability and behavioral plasticity. Finally, this study documents how the presence of humans, their artefacts and their activities facilitate the development of certain behavioral traditions in free-ranging nonhuman populations, thus providing valuable insights into how human–alloprimate relations can be restructured within the increasingly resource-competitive environments of the Anthropocene.

## Introduction

The aggressive pace of ecological niche construction by humanity has been permanently altering the natural environments of this planet. Such anthropogenic environmental modifications have brought in different kinds of novel selection pressures influencing the co-evolution of human and nonhuman populations that inhabit such disturbed environments, including the human–alloprimate interface (Fuentes, 2010). The almost-exclusive presence of human-oriented features at these interfaces pose various challenges for the nonhuman cohabitants of the interface, especially in comparison to their natural habitats (Lowry et al., 2013; Barrett et al., 2019).

Nonhuman primates appear to be desperately struggling for coexistence at the dramatically changing human–alloprimate interface, with more than 75% of nonhuman primate species confronting drastic population declines, habitat fragmentation, and local extinctions (Estrada et al., 2017). To cope with the challenges faced in such human-modified environments, behaviorally adaptable individuals in several nonhuman primate populations have now been shown to adopt novel behavioral responses, such as innovative food extraction techniques, modulated by their day-to-day experiences of interacting with novel environmental stimuli, including humans and their artefacts (Gruber et al., 2019). At human–alloprimate interfaces, for example, certain nonhuman primates incorporate human-sourced food items into their diet (Watanabe, 1989; Sinha et al., 2005; Sinha and Vijayakrishnan, 2017) and occasionally acquire such food through innovative, manipulative foraging techniques (McLennan and Hockings, 2014; Luncz et al., 2017; Dhananjaya et al., 2022) or even novel intentional communicative strategies (Deshpande et al., 2018).

The ‘extractive foraging’ hypothesis proposes that the availability of food is a major factor driving the propensity of nonhuman primates to develop such complex manipulative behaviors and that such ‘ecological necessity’ may lead to the evolution of tool use (Fox et al., 1999). Chimpanzees (*Pan troglodytes*) of Bossou, Guinea, for example, show an increase in tool-aided extractive foraging techniques during seasons of food scarcity (Yamakoshi, 1998). In contrast, the ‘ecological opportunity’ hypothesis proposes that prolonged exposure to favourable environmental conditions and repeated exposure to hard-to-extract food items may themselves promote the emergence and maintenance of complex extractive foraging behaviors in certain nonhuman populations, such as the chimpanzees of Gombe, Tanzania, who engage in tool-assisted termite-fishing only during the wet season, coinciding with high termite availability and accessibility (McGrew et al., 1979).

The two most important drivers of behavioral innovation, especially in the domains of foraging and feeding – necessity and opportunity – are both typically provided for by anthropogenic environments. The lack of natural food items, which often results from anthropogenic habitat modifications, may have made it necessary for these animals to explore human-source food items. Repeated interactions with humans and anthropogenic artefacts could have then provided them with opportunities to carry out such innovative explorations. Our empirical understanding of the nature and extent of such responses and more importantly, of the mechanisms underlying them, however, remains limited (Luncz et al., 2017; Gruber et al., 2019). The ‘affordance learning’ hypothesis, for example, proposes that innovative extractive foraging behaviors could derive from pre-existing schemata (Parker and Gibson, 1977). The regular interactions with an object – a hard-to-extract food item, for instance – could provide opportunities to appreciate the affordance of its physical and action-relevant properties, and finally contribute to the development of novel extractive foraging behaviors (Lockman, 2000; Mosley, 2021).

The ontogeny and evolution of stable, human-induced, behavioral, cognitive and cultural changes in nonhuman primate populations, we suggest, could be driven by several processes. These could potentially include, for example, individuals exploring specific functional relationships between different ecological components of anthropogenic environments, the development of innovative foraging techniques by animals in such situations, and the cognitive abilities of these individuals to learn and use the affordances of certain anthropogenic artefacts. Moreover, such innovative foraging behaviors, possibly mediated by mechanisms underlying phenotypic plasticity, once practiced regularly, could lead to the establishment of novel behavioral traditions in these primate populations through social learning (Watanabe, 1989; Sinha, 2005). A wide range of studies have thus highlighted the significant variability and flexibility in socially transmitted extractive foraging behaviors, now evident across populations, groups or even individuals (Whiten et al., 1999; van Schaik et al., 2003; Gumert et al., 2011; Lea et al., 2020).

The observed variation in tool-aided, extractive foraging behaviors across populations, in particular, appear to be most strongly driven by ecological necessity, opportunity, and the relative profitability of displaying such strategies (Yamakoshi, 1998; Sanz and Morgan, 2013; Koops et al., 2014). Sexual dimorphism and sex-based variation in energy requirements appear to have resulted in differences between males and females in tool-assisted foraging efficiency (Boesch and Boesch, 1984; Lonsdorf et al., 2004; Gumert et al., 2011). Age differences have also been able to explain within-group variation in complex foraging techniques; immature and less-experienced individuals, for instance, showed lower proficiency than did mature and more experienced ones, highlighting the slow development of extractive foraging skills through social learning and/or individual learning *via* trial-and-error processes and regular practice (Lonsdorf, 2005; Gunst et al., 2010; Schuppli et al., 2016). Similarly, inter-individual variation is expected to appear during the spread of human-induced innovative extractive foraging behaviors within social groups, especially of primates, who are known to be strikingly behaviorally innovative. Research on the pathways and patterns of diffusion of novel extractive foraging behaviors across fragmented and anthropogenically impacted populations could, therefore, provide unique opportunities to understand the evolution and development of behavioral innovations in social animals, particularly nonhuman primates.

In this study, we investigated one such innovative extractive foraging behavior – hereafter, bottle manipulation or bottle handling – in a population of bonnet macaques (*Macaca radiata*), living in an anthropogenic landscape. Although the range of this endemic macaque species is restricted to within peninsular India, it is widely found across varieties of habitats – from mountainous rainforests to dry deciduous scrubland, and from rural agricultural landscapes to completely urban settings (Sinha, 2001). With their high behavioral adaptability and flexibility, these commensal macaques have become one of the most successful cohabitants of humans, especially in urban environments. In addition to their remarkable phenotypic plasticity, a set of extraordinary cognitive abilities have aided these macaques to adopt innovative behavioral patterns that contribute to their survival across anthropogenic habitats of southern India (Sinha, 1997, 1998, 2005, 2017; Sinha and Mukhopadhyay, 2013; Sinha and Vijayakrishnan, 2017; Deshpande et al., 2018).

Bottle manipulation is one such innovative extractive foraging technique, used by these macaques to extract the contents of a bottle, first reported in two urban-dwelling groups of bonnet macaques in the city of Mysore (Mangalam and Singh, 2013). In this manipulative behavior, exhibited only in human-modified environments, an individual first acquires a plastic bottle – often filled with water, juice, or other human-sourced liquid food items – then engages in a series of bottle-directed handling techniques, as, for example, puncturing the container or removing the cap to extract the liquid contents and finally drinks the liquid. Mangalam and Singh (2013) found that 64% of sampled individuals succeeded in bottle manipulation but the success was biased towards females. Moreover, the techniques used to extract the fluid varied across individuals, with only 18% of the successful cases involving cap removal.

We had earlier observed bottle manipulation by individuals of different age and sex classes in multiple groups of bonnet macaques during a preliminary study of object manipulation in the same population (Pal, unpublished data). If such a novel behavior had indeed spread socially within the population, an exploration of its diversity and distribution over time could further our understanding of the adoption of innovative behavioral strategies by this species at the human–alloprimate interface. Moreover, a quantitative study of bottle-directed manipulation could help us identify the causal factors underlying behavioral innovation and diffusion within a macaque population. Our objective was thus three-fold: (a) document the distribution and diversity of bottle manipulation across individuals in this population of synurbic bonnet macaques, (b) investigate the intra-individual behavioral flexibility of the manipulative techniques directed towards this particular type of objects, and (c) explore the social and environmental components of this behavioral practice. To achieve these goals, we specifically tested the effects of three socio-demographic variables – group membership, age, and sex – and of two environmental variables – bottle availability and food provisioning by humans – on the frequency, form, and success of bottle manipulation by this population of macaques.

## Materials and methods

### Study site, species, population, and groups

This study was conducted on a population of free-ranging bonnet macaques inhabiting the Chamundi hill (76.671° E, 12.273° N) in the southern state of Karnataka, India. The top plateau – 1,024-1,054 m in height – of this isolated hillock system is surrounded by seven small hillocks, ranging between 861 and 944 m, and covering a total area of c. 17 km^2^. The Chamundeswari temple complex, one of the famous tourist destinations of southern India, is situated at the summit of this plateau. The plateau is heavily crowded, with a daily footfall of about 10,000 visitors to the temple complex, a neighbouring settlement of 150 houses and more than 200 tourism-related shops. The rest of the area is covered by natural dry deciduous and thorn-scrub vegetation, except a hill road that runs across the hill, from the foothills to the temple complex.

During a preliminary study, a total of seven groups of bonnet macaques were identified within the Chamundi hillock complex. After considering the accessibility of the habitat, four groups, namely DV, FH, PL, and RS (Table 1), widely distributed on the hillock and living under different kinds of anthropogenic pressures, were selected for the study. Among them, DV and PL were neighbouring groups, with overlapping home-ranges in and around the temple complex. While the movement of the DV group was mostly concentrated within the temple complex, the PL group ranged in the outer scrublands and occasionally visited the eastern periphery of the temple complex. The FH group was situated in the foothill region and ranged around the roadside human settlements, whereas the RS group was distributed midway on the hill road, between the foothills and the plateau. Although the four groups occupied the same human–macaque interface, the intensity of anthropogenic activities and frequency of human–macaque interactions varied across the groups, with the occurrence of human–macaque interactions being higher for the DV and FH groups than for the PL and RS groups.

**Table 1:** Demographic composition of the four study groups of bonnet macaques during Task I.

Group name	Group size	Adult male	Adult female	Subadult male	Juvenile male	Juvenile female	Infant	Group size, without infants
DV	42	10	11	5	6	3	7	35
FH	51	9	13	6	8	6	9	42
PL	25	4	7	5	3	3	3	22
RS	19	2	4	4	3	3	3	16
Overall	137	25	35	20	20	15	22	115

### Data collection

The study was part of a larger research project, conducted between September 2020 and March 2021, aiming to document the repertoire of object-oriented manipulative behaviors of free-ranging bonnet macaques in an anthropogenic landscape. Along with other forms of object manipulation, several individuals of the Chamundi population were observed to open plastic bottles and drink their contents.

In the first phase of the study, conducted between September and October 2020, we focused on the DV group, with ~250 h of observation over 40 days, and opportunistically recorded a total of 14 events of spontaneously expressed bottle manipulation by members of the group. We observed that several individuals of different age-sex classes were capable of engaging in various manipulative techniques to extract liquid materials from bottles. Approximately 75% of these bottle-manipulation events involved unsealed, capped, and partly filled one-litre plastic water bottles, most commonly available in the area. Based on the observations during the first phase of the study, we designed two field experimental tasks to further observe and analyze the different bottle-directed manipulative techniques in the study population.

### Task I: Distribution and diversity of bottle-directed manipulative techniques across the four study groups

Prior to initiating Task I, each of the four study groups were followed for 20 days to habituate and identify the study individuals. During this period, data on group movements and all bottle-manipulation events were collected through *ad libitum* sampling (Altmann, 1974). Each group member was categorized into one of the following six age-and sex classes: adult male, adult female (including subadult females), subadult male, juvenile male, juvenile female, and infant. Subadult females were included within the category of adult females as it is often morphologically difficult to discern the transition of a subadult female into adulthood in bonnet macaques, as it is for several other cercopithecine primates. As no infant was observed to independently engage in bottle manipulation during the first phase of our study, we excluded infants from all further investigations.

In Task I, we attempted to partially replicate the natural conditions of bottle manipulation in each of the four study groups; these groups were already familiar with the bottles and to human presence. We collected used, one-litre, water bottles from the local shops around the temple complex. These transparent, polyethene-terephthalate (PET) bottles had a wall thickness of 0.01 cm, dimensions of about 28 cm × 10 cm × 7.5 cm and a blue screwcap of circumference 9.5 cm. They all had sealing threading of ~135°, implying that less than one complete rotation was required to uncap these bottles. After procuring the bottles, we cleaned them, poured ~50 ml of fruit juice, obtained from local shops and familiar to the macaques, as reward for them, and re-capped them. Each bottle was manually re-capped by a single experimenter – AP – who employed a comparable force to maintain consistency in the tightness of the capping.

The experiments on bottle manipulation in Task I were conducted between 9:00 and 12:00 h on select days between November 2020 and January 2021 by the same two experimenters, AP and SM, who had earlier followed the study groups during the pilot study, as well as habituated and identified the individuals in these groups. A trial consisted of placing a bottle on the ground about 5 m away from a prospective focal individual, when no other group members were within 3 m of them. Each bottle-manipulation event was video-recorded with a Sony HDRCX405 (Sony, Tokyo, Japan) digital camcorder; we began the recording immediately after the focal individual began to approach the bottle, thus allowing us to record the entire interaction sequence. We terminated the recording after the focal individual moved away from the bottle or did not interact with it for 30 s. The trials were also ended if the focal individuals did not interact with the bottle for 120 s after the bottles were placed about 5 m of them. To examine behavioral variability in bottle-directed manipulative techniques across age-sex classes, both within and across groups, we maximized sampling diversity by selecting a new focal subject for each trial. Occasionally, due to spatial proximity and the close dominance ranks among particular group members, certain untargeted individuals would engage in the task before the focal subject could approach or take possession of the bottle. We excluded such cases and only considered those events in which a single individual engaged in bottle manipulation, right from the first approach until it voluntarily left the bottle, without any interference from other group members.

The four study groups comprised 137 individuals (115, excluding infants), with group sizes ranging from 19 (RS group) to 51 (FH group, Table 1). The trials were conducted for 24 days, with an average of 6 days/group and a range of 4–8 days. We recorded a total of 123 bottle-manipulation events, involving 74 individuals across the four groups, comprising 60% of the study individuals, with sampling proportions ranging from 57% (RS group) to 77% (FH group) of all individuals, excluding infants (Table 2). A bottle-manipulation event was considered successful if the focal subject engaged in some form of bottle manipulation that led it to extract the liquid from the bottle and drink it, thereby effectively solving Task I.

**Table 2:** Individuals (excluding infants), sampled during Task I, across the four study groups of bonnet macaques.

Group name	Sampled individuals	Adult male	Adult female	Subadult male	Juvenile male	Juvenile female	Total number of trials	Percentage individuals sampled	Mean and range of number of trials per individual
DV	21	6	8	3	3	1	32	60.00	1.52, 1–3
FH	24	7	11	3	0	3	30	57.14	1.25, 1–4
PL	17	4	6	3	2	2	31	77.27	1.82, 1–3
RS	12	2	4	4	1	1	30	75.00	2.50, 1–4
Total	74	19	29	13	6	7	123	60.16	1.66, 1–4

### Task II: Intra-individual behavioral flexibility and inter-individual variability in bottle-directed manipulative techniques

The DV group, which was the most accessible of all the study groups, had contributed the largest number of samples to Task I and had displayed a high proficiency (97%) of successful bottle manipulation, was thus selected to collect data on between-individual behavioral variability in bottle-opening techniques in Task II. As one of our objectives was also to examine the intra-individual behavioral flexibility of successful bottle manipulation, a different type of PET bottle was presented multiple times to selected individuals of the DV group during Task II. Although they were similar in size to the bottles used in Task I, with dimensions of 27 cm × 11 cm × 10 cm, these bottles were semi-transparent, had a thicker wall of 0.1 cm, a bigger cap with a circumference of 15.5 cm, and a long screw-neck, which required two complete rotations – 360° × 2 – to uncap it. These bottles thus presented more-constrained, cap-directed affordances for the macaques, due to their larger caps with longer screw-necks.

A total of 10 subject macaques of different age-sex classes – five adult males (BHA, MO, SALU, SRP and TUMOR), three adult females (RUBY, JBE, and LED), one subadult male (LEP) and one juvenile male (JAM) – who had successfully performed Task I, were selected for Task II. The task was presented six times to each of these subjects, with a time interval of >2 days between two successive presentations, to compare the intra-and inter-individual variability of bottle-opening techniques. The task-presentation procedure was identical to that of Task I, with only those events in which a single individual engaged in bottle manipulation, from the first approach until it voluntarily left the bottle, without any interference from other group members, being considered for analysis.

### Bottle availability and food provisioning

Data on the availability of discarded bottles in the study area and the frequency of food provisioning to all four study groups were collected as proxy variables to examine the impact of environmental opportunities on the diversity and distribution of bottle manipulation in the study population.

During the Task I experiments, which were conducted between 9:00 and 12:00 h on select days between November 2020 and January 2021, the number of plastic bottles available to each of the four study groups within a 30-m radius of each group centre – the group centre being defined as the site of the largest aggregate of individuals at a given time – was recorded every hour but only during the duration of the experiment. This naturally meant that there was a minimum of zero to a maximum of three data points of bottle availability per day of experimentation. The availability of bottles was then calculated as number of bottles/ha or 10,000 m^2^, averaged over the first 15 data points for each of the study groups. If two consecutive group centres for a group were < 50 m apart, the second data point was not recorded, and a resampling was conducted an hour later. As the bottles were stationary objects and were cleaned twice a week by municipal workers, we maintained an interval of >7 days between two successive sampling days, all groups considered.

The macaques in the temple complex were regularly provisioned with food by the devotees and tourists visiting the temple, with most of the provisioning occurring between 09:00 and 13:00 h along the roads leading to the temple. Data on provisioning of the four study groups were thus collected in terms of events of provisioning – defined as every occasion when a food item was provided to an individual or a group of macaques by a human – received by a group per hour. We recorded the data on provisioning by focal group sampling of 30-min duration on each troop between 09:00 and 13:00 h, but either before or after the Task I experiment was conducted, on each day of experimentation. A mean total sampling time of 15.88 h (range of 15 to 17 h) was conducted per group, the data being collected across a mean of 15 (range of 14 to 16) days for each group.

### Data extraction

Each recorded video clip from Task I was played twice, once at normal speed and then once at a slower speed (0.5X), in the VLC Media Player (3.0.16). If needed, we zoomed (4X) into the video segments that required better visibility. The data extracted from these video clips included group name, age-sex class of the focal subject, ID of the focal subject, bottle-directed manipulative techniques, duration of bottle-manipulation events, and outcome – success or failure – in the extraction of the liquid reward. All the bottle-directed manipulative techniques found in the entire Task-I data set were then defined, and an ethogram of bottle manipulation constructed (Table 3; Supplementary material 1). These bottle-directed manipulative techniques were broadly classified into two categories: (a) bottle-opening and (b) liquid-drinking. Cap-directed manipulative techniques were identified independently to examine the familiarity of macaques with the screw-cap mechanism.

**Table 3:** Ethogram and distribution of different manipulative techniques, employed within each bottle-directed behavioral category, by the study groups of bonnet macaques during Tasks I and II.

Behavioral category	Definition of techniques employed	Technique code	Nature of technique	Whether performed in	
				Task I	Task II
Bottle-opening	Biting the body of the bottle	BBB	NS	Yes	Yes
	Biting the cap of the bottle	BCB^C^	NS	Yes	Yes
	Biting the heel of the bottle	BHB	NS	Yes	Yes
	Biting the neck of the bottle	BNB	NS	Yes	Yes
	Holding the bottle with one or both hand(s)	HOB	NS	Yes	Yes
	Rolling the horizontally placed bottle on the ground	RGB	NS	Yes	Yes
	Rolling the bottle between both hands while lifting it with one or both hand(s)	RHB	NS	Yes	Yes
	Smelling the bottle cap or the bottle opening after removing the cap	SNF	NS	Yes	Yes
	Removing the cap by rotating it with one hand while holding the bottle horizontally with the other hand and/or leg(s)	HHB^C^	S	Yes	Yes
	Loosening the cap with mouth while holding the cap and shoulder of the vertically placed bottle with both hands	HHM^C^	S	Yes	Yes
	Holding the cap in the mouth and rotating the vertically placed bottle with both hands	HMB^C^	S	Yes	Yes
	Removing the cap by rotating it with one hand while holding the bottle vertically with the other hand and/or leg(s)	VHB^C^	S	Yes	Yes
Liquid-drinking	Licking up accidentally spilt liquid from the ground	ASB	NS	Yes	Yes
	Holding the horizontally placed bottle on the ground and drinking liquid from a punctured opening	HDL	NS	Yes	No
	Pouring the liquid from the bottle onto the ground and licking/drinking it up	IPB	NS	Yes	Yes
	Licking the spilt liquid from the bottle opening after loosening the cap	LBB	NS	No	Yes
	Licking the inside of the cap after removing it from the bottle	LCB	NS	No	Yes
	Holding the bottle above the mouth with both hands and/or leg(s) and drinking liquid from the opening	HDC	S	Yes	Yes
	Holding the bottle with a punctured opening above the mouth with both hands and drinking from it	HDP	S	Yes	Yes

S, Sophisticated behavioral technique; NS, Non-sophisticated behavioral technique; ^C^, Cap-directed manipulative technique.

Moreover, the bottle-directed manipulative techniques were also classified, according to their efficiency, into two classes: (a) sophisticated techniques, defined as the relatively more efficient, successful opening of the bottle by removing the screw-cap and effectively drinking its contents, without any spilling, similarly to how humans do it, and (b) non-sophisticated techniques, involving the forceful manipulation of the bottle, with relatively inefficient liquid-drinking techniques. We observed six sophisticated bottle-directed, behavioral techniques, including four pertaining to opening bottles – HHB, HHM, HMB, and VHB – and two pertaining to drinking the liquid within – HDC and HDP (Table 3).

Data from the Task-II video clips – including the nature and number of bottle-directed, manipulative techniques and whether they were sophisticated or not – of all six events of each focal subject, were similarly extracted. If, during a bottle-manipulation event, a focal subject was able to extract the liquid from a bottle and drink it, irrespective of the bottle-opening and liquid-drinking techniques employed and independently of whether the techniques were sophisticated or not, the bottle-manipulation event was considered successful.

### Statistical analysis

We employed logistic regression analyses to examine the distribution of bottle-directed manipulative techniques and successful bottle manipulation events across three age classes: adult, subadult and juvenile, and two sex classes: male and female, in the four study groups, DV, FH, PL and RS. In Task I, five dependent variables were scored as binary classes – presence/absence or 1/0 – of bottle-manipulation success, sophisticated bottle-directed techniques, sophisticated bottle-opening techniques, sophisticated liquid-drinking techniques, and whether the first contact with a bottle was a cap-directed manipulative technique or not. These dependent variables were tested against three explanatory variables: group membership, age class, and sex class. Analysis of Variance (ANOVA) was employed to examine the variability in bottle-directed manipulative techniques across the study groups and across subjects for Tasks I and II, respectively.

To test for differences in bottle availability and frequency of food provisioning across the four study groups, we used the Kruskal-Wallis rank-sum test. The possible relationships of bottle availability and food-provisioning frequency with bottle-manipulation success, frequency of sophisticated bottle-directed techniques, and cap-directed manipulative techniques were examined using the Spearman’s rank correlation test.

To examine inter-individual variability in bottle manipulation across the study groups, we used the presence/absence data, obtained from the five aforementioned dependent variables in Task-I events, along with group membership, age-and sex categories that were included as grouping variables. We performed Non-metric Multi-Dimensional Scaling (NMDS) on this dataset, using the Jaccard similarity measure, to characterize the bottle-directed manipulative techniques, observed in this task, in terms of their distribution around and distance from the mean values of the three grouping variables of the study population. Thereafter, Analysis of Similarities or ANOSIM was performed to examine the mean rank dissimilarities between the study groups and the three grouping variables, namely group membership, age-and sex categories, all tested independently.

For Task II, we used Analysis of Variance or ANOVA to test inter-individual variability in bottle manipulation and problem-solving time, defined as the time interval between the first contact with the bottle and use of the first liquid-drinking technique. To examine intra-individual flexibility and inter-individual dissimilarities in bottle-directed manipulative techniques, we used NMDS and the Jaccard similarity measure, after transforming the data on bottle-directed manipulative techniques into binary classes and using individual ID as a grouping variable. Thereafter, differences in intra-and inter-individual mean rank dissimilarities in manipulative techniques were tested by ANOSIM.

All statistical analyses were performed using R statistical language V4.1.0 with R Studio IDE for R V1.4.113. We used ‘vegan’ – Community Ecology Package, Vegan 2.4.3 – for NMDS and ANOSIM.

## Results

### Distribution and diversity of bottle-directed manipulative techniques amongst bonnet macaques

All the sampled individuals, belonging to all age-sex classes, except infants, across all four study groups were observed to engage in bottle manipulation during the study period. Apart from acquiring partially filled bottles from dustbins and roadsides, individuals were observed to surreptitiously take away new, sealed bottles from local shops, as well as display both aggressive and non-aggressive approaches towards tourists to obtain the bottles they were carrying or drinking from. During the long-term follow of the DV group, we recorded the migration of six individuals – four adult and two subadult males – into the group. Three of these four immigrant adult males were also seen to successfully extract liquid contents from the bottle, while two of them occasionally used sophisticated behavioral techniques to extract the bottle contents.

Of the 19 bottle-directed manipulative techniques displayed by the study groups (Table 3), 17, including all 12 bottle-opening but only five of the seven liquid-drinking, techniques were performed by the study subjects of all the groups during Task I. We observed (1) a total of five cap-directed manipulative techniques, including four sophisticated – HHB, HHM, HMB, and VHB – techniques, wherein the macaques removed the cap of the bottle by rotating it with the help of their limbs and mouth, and (2) a non-sophisticated – BCB – technique, wherein the cap was removed by forceful biting. Amongst the study groups, 12 and 11 bottle-directed manipulative techniques were recorded in the DV and FH groups respectively, and 14 such techniques each in the PL and RS groups. The number and distribution of these techniques, exhibited by the study groups across all the events of Task I are shown in Table 4.

**Table 4:** Distribution of bottle-directed manipulative techniques and events across the four study bonnet macaque groups in Task I.

Bottle-directed manipulative techniques		Study groups				Total
		DV	FH	PL	RS	
Number of events		32	31	30	30	123
Number of techniques		12	11	14	14	17
Mean number of techniques per event		3.19	2.53	2.52	2.83	2.77
**Number of events that involved specific techniques**						
Bottle-opening	BBB	0	0	5	7	12
	BCB	1	7	8	13	29
	BHB	2	1	15	9	27
	BNB	0	1	2	6	9
	HHB	4	2	5	1	12
	HHM	8	0	1	0	9
	HMB	6	9	3	0	18
	HOB	0	0	8	3	11
	RGB	3	1	0	9	13
	RHB	2	2	0	2	6
	SNF	18	10	3	8	39
	VHB	14	13	2	0	29
Liquid-drinking	ASB	12	0	2	4	18
	HDC	21	25	7	7	60
	HDL	0	0	0	5	5
	HDP	0	2	11	6	19
	IPB	11	3	6	5	25

Note that each event could have involved the use of more than one technique.

Of the bottle-opening techniques, the relatively simple techniques of BCB and BHB were commonly observed in the population, especially in the PL and RS groups though the DV and, to a certain extent, FH groups displayed relatively low levels of these behaviors. In contrast, the relatively sophisticated manipulative techniques of HMB and VHB were extensively used by the members of the DV and FH groups. The DV group also exhibited comparatively high frequencies of the other sophisticated behavioral techniques of HHB and HHM (Table 4).

HDC was the most frequently observed liquid-drinking technique, employed in ~66% of all events that involved bottle manipulation by the DV group, ~81% by the FH group and ~ 49% overall (Table 4). It is perhaps noteworthy that one of these techniques, HDL, was uniquely recorded during five bottle-manipulation events in the RS group alone.

The mean number of bottle-directed manipulative techniques, displayed per event during Task I, was 2.77 ± 0.90_SD_, which significantly varied across the study groups (ANOVA, F_3, 119_ = 4.21, *p* < 0.01). The variability in the extent to which such techniques were exhibited, however, was significantly higher in the DV group than in the FH (post-hoc Tukey’s HSD test, *p* = 0.02) or in the PL (*p* = 0.01) groups.

The mean group rate of successful bottle-manipulation events, measured as the proportion of Task-I events (± SE) that were solved by the focal subjects across the four macaque groups was 0.94 (± 0.06, N = 123), with all the groups performing at comparable levels (Table 5).

**Table 5:** Proportion of successful bottle-manipulation events and sophisticated techniques within different bottle-directed behavioral categories, displayed by different age-sex classes in the four study bonnet macaque groups during Task I.

Behavioral category	Group				Age class			Sex class		Overall
	DV	FH	PL	RS	Adult	Subadult	Juvenile	Female	Male	
Successful bottle manipulation	0.97	1.00	0.87	0.90	0.99	0.92	0.72	0.98	0.90	0.94
Sophisticated bottle-opening techniques	0.94	0.80	0.35	0.33	0.57	0.58	0.33	0.48	0.58	0.53
Sophisticated liquid-drinking techniques	0.91	0.94	0.43	0.40	0.69	0.83	0.33	0.63	0.70	0.67
Overall sophisticated techniques	0.97	0.93	0.42	0.40	0.69	0.83	0.44	0.63	0.73	0.68
First contact: Cap-directed	0.97	0.97	0.55	0.43	0.74	0.92	0.44	0.68	0.78	0.73

Logistic regression analysis revealed that the subject macaque’s age class had a statistically significant effect on the probability of successful bottle manipulation in Task I, with juveniles being less successful than subjects from other age classes. Group membership and sex class did not, however, have a pronounced effect (Table 6).

**Table 6:** Logistic regression analysis of the determinants of successful bottle manipulation and the occurrence of sophisticated bottle-directed techniques across all age-sex classes in the four study macaque groups during Task I.

Dependent variable	Independent variable	Estimated coefficient	SE	Wald’s *Z*	*p*	Pseudo-*R*^2^
Successful bottle manipulation	Group: FH	15.10	2.87	0.01	1.00	0.44
	Group: PL	−2.50	1.52	−1.64	0.10	
	Group: RS	−2.84	1.68	−1.70	0.09	
	**Age: Juvenile**	**−4.49**	**1.51**	**−2.98**	**< 0.01**	
	Age: Subadult	−1.28	1.28	−1.00	0.32	
	Sex: Male	−2.83	1.49	−1.89	0.06	
Sophisticated bottle-opening techniques	Group: FH	−1.67	1.01	−1.66	0.10	0.57
	**Group: PL**	**−4.32**	**1.03**	**−4.19**	**< 0.001**	
	**Group: RS**	**−7.77**	**1.46**	**−5.33**	**< 0.001**	
	**Age: Juvenile**	**−2.44**	**0.92**	**−2.67**	**< 0.01**	
	Age: Subadult	−0.15	0.91	−0.16	0.87	
	**Sex: Male**	**2.03**	**0.79**	**2.57**	**0.01**	
Sophisticated liquid-drinking techniques	Group: FH	0.13	1.10	0.12	0.09	0.39
	**Group: PL**	**−3.49**	**0.94**	**−3.70**	**< 0.001**	
	**Group: RS**	**−4.09**	**0.97**	**−4.20**	**< 0.001**	
	**Age: Juvenile**	**−2.85**	**0.90**	**−3.15**	**< 0.001**	
	Age: Subadult	1.04	0.74	1.40	0.16	
	Sex: Male	0.65	0.58	1.12	0.26	
Overall sophisticated techniques	Group: FH	−0.99	1.33	−0.74	0.46	0.42
	**Group: PL**	**−4.51**	**1.22**	**−3.69**	**< 0.001**	
	**Group: RS**	**−5.11**	**1.26**	**−4.05**	**< 0.001**	
	**Age: Juvenile**	**−2.24**	**0.92**	**−2.43**	**0.02**	
	Age: Subadult	0.89	0.74	1.20	0.23	
	Sex: Male	1.04	0.62	1.67	0.10	
First contact: Cap-directed	Group: FH	−0.27	1.56	−0.17	0.87	0.47
	**Group: PL**	**−4.36**	**1.39**	**−3.14**	**< 0.01**	
	**Group: RS**	**−5.68**	**1.45**	**−3.91**	**< 0.001**	
	**Age: Juvenile**	**−3.11**	**1.13**	**−2.75**	**< 0.01**	
	Age: Subadult	1.68	0.92	1.83	0.07	
	Sex: Male	0.88	0.64	1.37	0.17	

Statistically significant relationships (*p* ≤ 0.05) are indicated in bold.

The proportion of sophisticated bottle-opening techniques exhibited per event varied widely across the groups, ranging between 0.33 in the RS group and 0.94 in the DV group. All the three independent variables tested – group membership, age class, and sex class – significantly affected the distribution of sophisticated bottle-opening techniques (Table 6) with relatively lower proportions being displayed by PL and RS group members and by juveniles in the study population; adult and subadult males, however, appeared to be particularly adept at these techniques (Table 5).

The proportion of sophisticated liquid-drinking techniques also varied widely across groups, with a range of 0.40–0.93 per event, as it did across age classes, with a range of 0.33–0.83 per event. Members of the PL and RS groups, as well as juvenile subjects, once again, displayed significantly reduced proportions of sophisticated liquid-drinking techniques (Table 5). Finally, the proportion of manipulative techniques that were primarily directed towards the bottle cap varied greatly across groups, with a range of 0.43–0.97 per event, and between age classes, with a range of 0.44–0.92 per event. Members of the PL and RS groups and juvenile individuals also exhibited significantly reduced tendencies to direct their initial approaches towards bottle caps than did the other group members and older subjects (Table 5).

**Figure 1:**
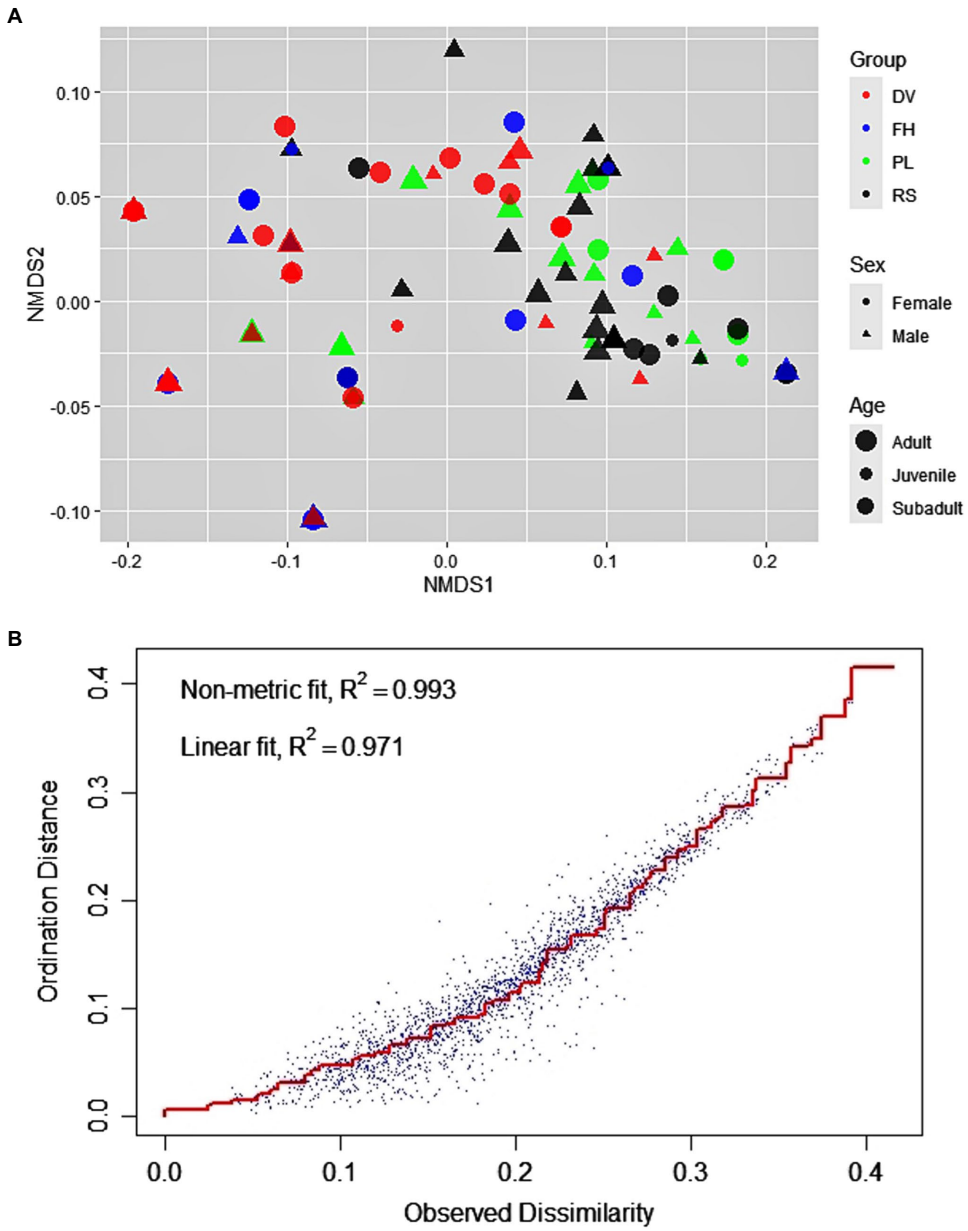
Distribution of the 123 events of bottle-directed manipulative techniques, displayed by the study bonnet macaque population during Task I, as analyzed by two-dimensional Non-metric Multi-Dimensional Scaling (NMDS), using the Jaccard similarity measure. (**A).** The groups are indicated by different colors: DV – red, FH – blue, PL – green, RS – black; sexes by different shapes: female – circle, male – triangle; and age classes by the size of the points: adult > subadult > juvenile. (**B).** Stress-plot of the NMDS. Stress = 0.08, non-metric R^2^ = 0.993.

**Figure 2:**
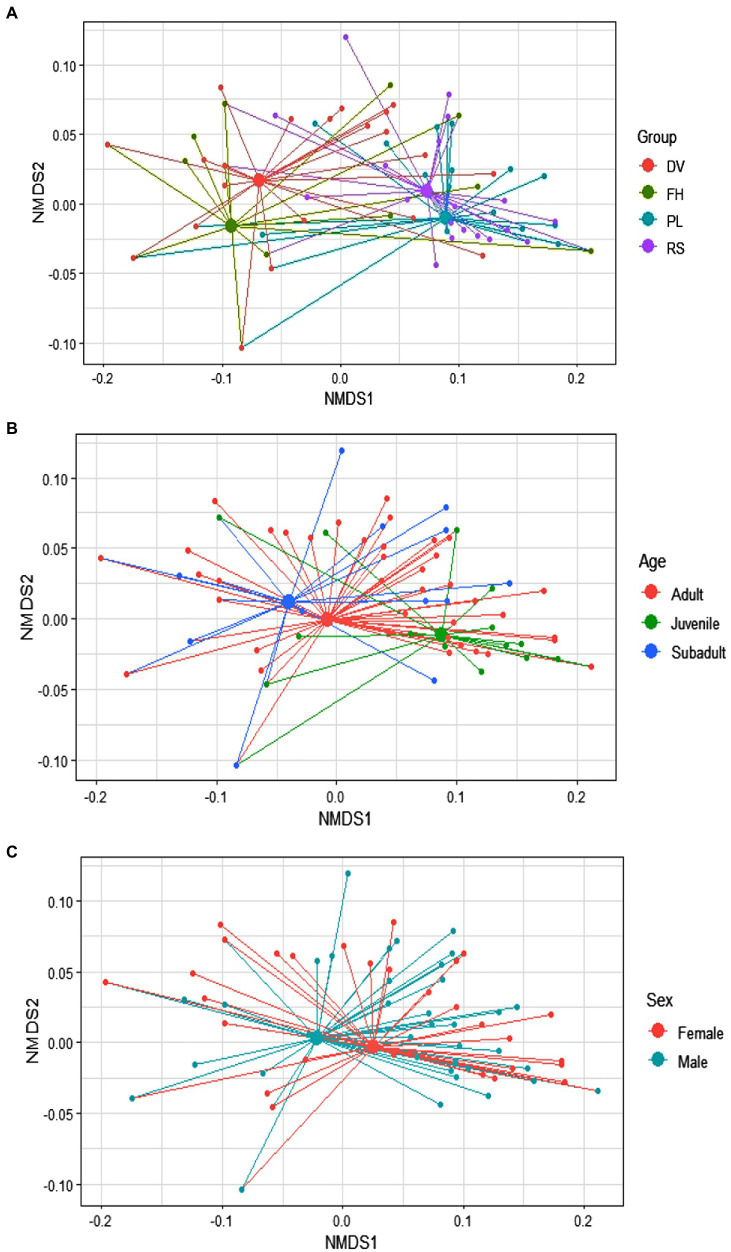
Variability of the 123 events of bottle-directed manipulative techniques, displayed by the study bonnet macaque population during Task I, as indicated by the distance of individual values (small solid circles) from the mean values (large solid circles) within the three considered variables of group membership **(A)**, age class **(B)**, and sex class **(C)**. These spider diagrams represent two-dimensional Non-metric Multi-Dimensional Scaling (NMDS) plots of the three variables, represented by different colors.

There was considerable variation in the use of bottle-directed manipulative techniques within the study population (Two-dimensional NMDS, using Jaccard similarity matrix; stress = 0.08; non-metric R^2^ = 0.993; Figures 1A,B). We found statistically significant dissimilarities in the employment of manipulative techniques across groups (ANOSIM, R = 0.23, *p* = 0.01) and between sexes (R = 0.03, *p* = 0.01), although the explanatory power of the model was relatively lower for sex-wise segregation (Figures 2A,C). The dissimilarities in manipulative techniques within age classes were, however, higher than that between them (R = 0.03, *p* = 0.20; Figure 2B).

### Bottle availability and food provisioning

Bottle availability was highest for the DV group (45.63 bottles/ha), followed by PL and FH (both 9.91/ha), and by the RS group (1.77/ha); these intergroup differences were statistically significant (Chi-square test, χ2 = 18.41, df = 3, *p* < 0.001). The macaques of the DV group were also the most frequently provisioned (0.80 events/h/group), followed by those of the FH (0.60/h/group), PL (0.33/h/group), and RS (0.07/h/group) groups, these differences once again being statistically significant (χ2 = 18.21, df = 3, *p* < 0.001). We found positive correlations between frequency of food provisioning and (1) frequency of use of sophisticated bottle-opening techniques (Spearman’s rank correlation, R_S_ = 0.99, *p* = 0.008), (2) overall frequency of cap-directed manipulative techniques (R_S_ = 0.96, *p* = 0.04), and (3) frequency of manipulative techniques that initially targeted bottle caps (R_S_ = 0.95, *p* = 0.05). There was, however, no statistically significant correlation between bottle availability and the frequency of these bottle-directed manipulative techniques across the study population, that is, when the four study groups were taken together (data not shown).

### Intra-individual flexibility and inter-individual variability

To solve Task II, the subject macaques of the DV group performed a total of 19 bottle-directed manipulative techniques, including two novel techniques for drinking liquid – LBB and LCB – not recorded during Task I (Table 3). VHB, HDC, and SNF were the three most common manipulative techniques, performed by nine of 10 focal subjects, whereas HMB was exhibited by only one individual.

When the 60 events of bottle opening were analyzed, the mean number of manipulative techniques displayed per event – 5.92 (± 2.78_SD_) – and the task-solving time, as monitored by the duration of the event – 29.24 (± 43.05_SD_) sec – varied significantly across the focal subjects (number of manipulative techniques per event: ANOVA, F_9, 50_ = 8.34, *p* < 0.01; task-solving time: F_9, 49_ = 4.17, *p* < 0.01). The number of manipulative techniques displayed by a subject macaque within an event also positively correlated to the duration of the event or the time taken to finally access the fluid within the bottle (Spearman’s rank correlation, R_S_ = 0.58, N = 60, *p* < 0.001).

**Figure 3:**
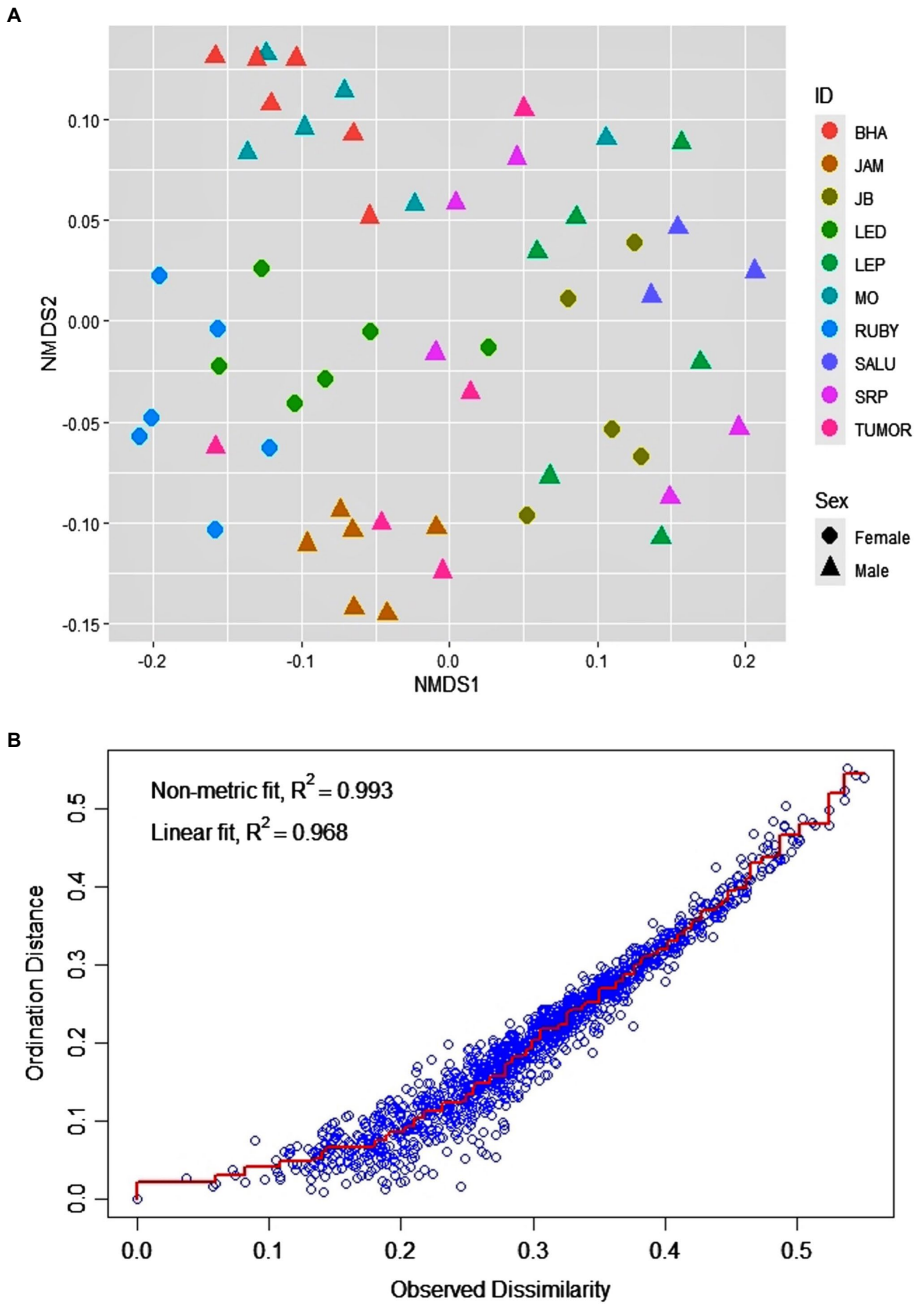
Distribution of the 60 events of bottle-directed manipulative techniques, displayed by the 10 study bonnet macaques of the DV group during Task II, as analyzed by two-dimensional Non-metric Multi-Dimensional Scaling (NMDS), using the Jaccard similarity measure. **(A).** Individual macaques are indicated by different colors and the two sexes by different shapes: female – circle, male – triangle. **(B).** Stress-plot of the NMDS model. Stress = 0.08, non-metric R^2^ = 0.993.

**Figure 4:**
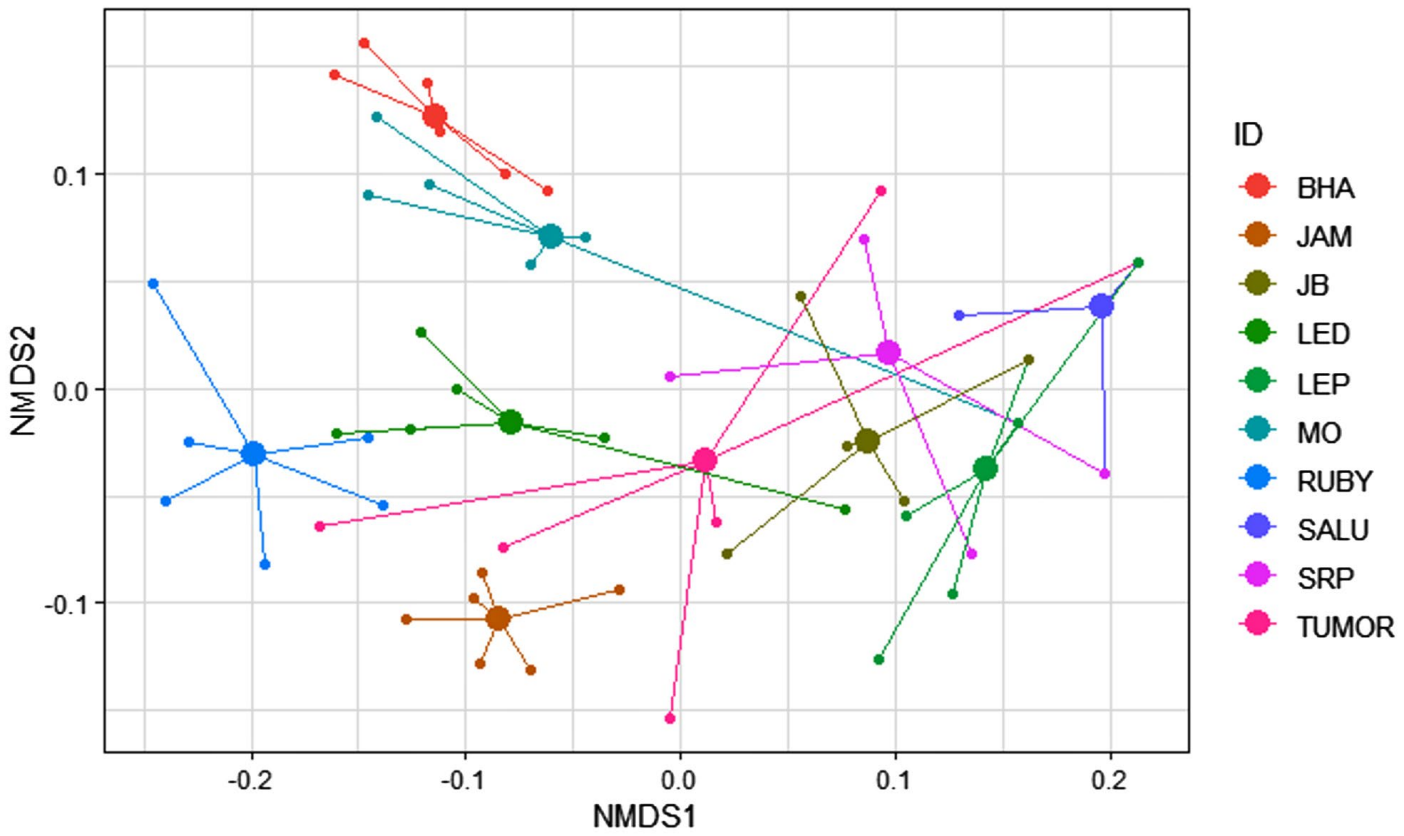
Variability of the 60 events of bottle-directed manipulative techniques, displayed by the 10 study bonnet macaques of the DV group during Task II, as indicated by the distance of each trial (small solid circles) from the mean value (large solid circles) of the six independent trials, conducted for each individual. This spider diagram represents a two-dimensional Non-metric Multi-Dimensional Scaling (NMDS) plot of all the 60 observed events, performed by the 10 individuals, represented by different colors.

The macaques of the DV troop showed significant inter-individual variation as well as a remarkable level of within-individual flexibility in their use of bottle-directed manipulative techniques during Task II, as revealed by two-dimensional NMDS, using a Jaccard similarity measure (stress = 0.09, non-metric R^2^ = 0.993; Figure 3). A close analysis of this variation, however, revealed, as could be expected, greater intra-individual similarities in manipulative techniques, as opposed to significantly higher levels of inter-individual variability within the study group (ANOSIM, R = 0.65, *p* = 0.01, Figure 4).

## Discussion

We recorded bottle-directed manipulative and extractive foraging behavior across individuals of different age-and sex classes – except infants – in our four study groups of synurbic bonnet macaques during the course of this study. Importantly, there was also a high rate of liquid-extraction success in this human-induced, foraging task, which all the focal individuals attempted to solve.

Two socio-demographic factors – age class and group membership – and one environmental factor – food provisioning – could be identified as major determinants of inter-individual variation in several aspects of the bottle-manipulative behavior exhibited, including first contact with bottles that were cap-directed, the sophisticated nature of the manipulative techniques performed and bottle-opening success. We also found high levels of intra-individual similarities and inter-individual differences within one of our study groups in the expression of bottle-handling techniques.

Our analysis revealed that age was a significant predictor of the frequency of sophisticated bottle-opening techniques and successful bottle manipulation. Older individuals performed more complex behavioral patterns and were more skilful at extracting the liquid than were younger individuals. These results suggest that age-related physical maturation, which could include enhanced bodily strength or dental development, and experiential, possibly trial-and-error, learning could be essential to the acquisition of foraging competence, pertaining to this particular task (Inoue-Nakamura and Matsuzawa, 1997; Gunst et al., 2010; Resende et al., 2014). Indeed, successful bottle manipulation, we believe, implies the execution of complex, multi-step, behavioral sequences, requiring perceptual learning, sensorimotor coordination and appropriate cognitive skills, including, but perhaps not restricted to, memory and associative learning. These psycho-physical characteristics may be gradually acquired by juvenile macaques through extended individual practice during their physical and intellectual maturation, ultimately culminating in mastery over sophisticated, bottle-manipulative techniques. It is noteworthy that these results could be explained by mechanisms earlier postulated to underlie the acquisition of other object-directed, manipulative techniques in nonhuman primates (Inoue-Nakamura and Matsuzawa, 1997; Gunst et al., 2010; Resende et al., 2014; Schuppli et al., 2016; Leca et al., 2021).

There are several lines of argument that potentially support the view that social learning could have also influenced the acquisition of bottle-manipulative techniques, exhibited by this macaque population. First, we found that group membership was a significant predictor of inter-individual variation in the first cap-directed contact with bottles, displayed by the study individuals and in their performance of both sophisticated bottle-opening and liquid-drinking techniques. Second, newly immigrated males appeared to adopt the bottle-associated extractive foraging techniques of their new host groups very rapidly, although this could have also been due to them having earlier learnt such manipulative techniques, either by individual trial-and-error learning or by social learning from their previous associations with conspecific individuals. Third, there were statistically significant dissimilarities in bottle-directed manipulative techniques across groups. Fourth, our study shows that bottle manipulation is now commonly practiced by individuals of all age groups in the Chamundi population of bonnet macaques, as compared to its rather limited distribution, restricted to only a few members of a single group of macaques, ranging in the nearby Chamundeswari temple complex, as documented in a recent study (Mangalam and Singh, 2013). Fifth, the relatively relaxed social relationships, prevailing in bonnet macaque societies even under regimes of enhanced resource competition (Ram et al., 2003; Sinha et al., 2005), are likely to promote social learning opportunities in such species (Coussi-Korbel and Fragaszy, 1995; van Boekholt et al., 2021). We thus argue that the current ubiquity and rapid spread of these innovative, extractive foraging techniques within and across several groups of this macaque population are probably more due to social transmission of these practices than to multiple, independent innovations across the population (*cf.* Sinha, 2005).

Intra-group social spread of food-related behavioral innovations have also been reported in different populations of monkeys and great apes (Whiten et al., 1999; Moura and Lee, 2004; Gumert et al., 2011). In a population of Japanese macaques *Macaca fuscata*, living on Koshima island, southwest Japan, sweet potato-washing behavior – another human-induced food-processing technique – was first performed by a juvenile female innovator and then socially transmitted to most of her group members within a decade, between 1953 and 1962, even though the early stages of propagation appeared to be biased toward younger individuals, with an initial horizontal transmission of the behavior amongst the innovator’s peer playmates (Kawai, 1965; Hirata et al., 2008). Similarly, after being first recorded in a single juvenile female innovator in a Japanese macaque population, living at Arashiyama, near Kyoto in mainland Japan, stone handling – an object-directed, versatile, largely non-instrumental and apparently playful, manipulative behavior – has also spread gradually across multiple generations of all matrilines, at least within one particular group (Huffman and Quiatt, 1986; Leca et al., 2012).

Even though bottle availability was not significantly associated with inter-group differences in bottle-directed manipulative techniques in our study population of bonnet macaques, certain obvious environmental determinants of the emergence and spread of this behavioral practice in the Chamundi population could potentially not be ruled out. The speed of diffusion of a novel foraging behavior across conspecific individuals is typically influenced by a set of ecological opportunities. Subtle changes in the local availability of plastic bottles, together with the exploratory propensities of bonnet macaques, a highly adaptable species, to acquire alternative subsistence resources from human-influenced environments, might have played an important role in the rapid and extensive diffusion of this behavioral practice. As discarded plastic bottles invariably result from human activities, the emergence of the bottle-directed, extractive foraging behavior, observed during this study, is certainly a by-product of anthropogenic disturbance of the environment. Once this behavioral innovation was stably established across different macaque groups, however, this behavioral trait appears to have become an integral part of the behavioral repertoire of this macaque population, thus providing an additional example of human-induced cultural change in a cercopithecine primate species (see also Leca et al., 2012, 2021; Gruber et al., 2019).

While the nature and extent of sophisticated bottle-opening techniques differed across our study groups, there was also a significant level of variability in the use of these techniques within groups. It was thus not surprising that group membership was not a significant predictor of bottle-manipulation success. To solve a bottle-manipulation task, an individual should process at least two pieces of information: (a) the bottle contains a drinkable liquid, and (b) this content can be extracted from the container by manipulation. The information that the bottle contains drinkable liquid may be acquired by observing skilled group members engaged in bottle manipulation or from own personal experience. As the liquid material can potentially be extracted by performing various bottle-directed manipulative techniques, a naïve individual could explore the bottle by exhibiting certain behavioral techniques in a series of attempts to extract its contents. These experiential processes of trial-and-error learning could have contributed to the striking inter-individual variability in bottle manipulation that we documented during our study.

To uncap a bottle, however, an individual would need a more detailed and sophisticated understanding of the complexity of the screw-cap mechanism, an alternative approach to solving the task by forcefully puncturing the bottle (*cf.* Mosley, 2021). This behavioral step could potentially be learnt through a combination of social and individual learning processes. The comparatively low distribution of first contacts with bottles that were cap-directed as well as of sophisticated manipulative techniques in the study PL and RS macaque groups could perhaps be best explained by relatively fewer opportunities (1) to encounter discarded plastic bottles, due to the generally lower availability of bottles in the habitats of these groups, and also (2) to observe a proficient group member performing these behaviors, once again due to the relatively low frequencies of food provisioning in these groups.

To efficiently drink liquid, extracted from a bottle while minimising the risk of spillage, an individual macaque needs to grasp information about the properties of fluids through regular interactions with fluid-containing bottles. A similar explanation, as above, could thus be invoked to explain the observed inter-group variation in the application of sophisticated liquid-drinking techniques. Nevertheless, in accordance with the ‘relative profitability’ hypothesis, postulated to explain the successful performance of a foraging task, an individual is expected to opt for the most energy-efficient techniques, to maximize trade-offs in energy expenditure (Rutz and St Clair, 2012; Sanz and Morgan, 2013). Having, therefore, lived in habitats with high human-induced environmental opportunities, members of the DV and FH macaque groups could have become proficient in the use of energy-efficient, bottle-directed, manipulative techniques. This, we believe, could explain the predominant distribution of sophisticated bottle-opening and liquid-drinking techniques, as well as of cap-directed first contacts with bottles, in these two groups, which were exposed to higher bottle availability and more frequent food provisioning than were the PL and RS groups, which primarily employed greater frequencies of non-sophisticated techniques to extract liquid from bottles.

It should be noted that such inter-group variation in socially transmitted, behavioral traits, such as extractive foraging, which we documented in this study, have been commonly observed in nonhuman primates, typically spurred by different, often specific, sets of ecological conditions, demographic structures, social organizations and psycho-cognitive mechanisms (van Schaik et al., 2003; Sanz and Morgan, 2013; Koops et al., 2014; Kaufhold and Van Leeuwen, 2019). Between-group variation in stone-assisted, hammering techniques to extract marine prey was, for example, observed in Burmese long-tailed macaques *Macaca fascicularis aurea* (Tan et al., 2015) while stone hammer selection to crack open nuts differed between neighbouring chimpanzee groups (Luncz et al., 2012). Although previous research has suggested ecological opportunities to be a strong force driving inter-group variation in complex manipulative behavior, most of those comparisons were focused on distant communities (McGrew et al., 1979; Yamakoshi, 1998; Sanz and Morgan, 2013; Koops et al., 2014). The study of differences in stone hammer selection across three neighbouring chimpanzee groups, in direct contrast, did not support the ‘ecological opportunity’ hypothesis, as all these groups lived under similar ecological opportunities, in which nut and hammer availability were equivalent across sites (Luncz et al., 2012). Surprisingly, despite living in the same habitat, with frequent inter-group interactions and migrations, our study bonnet macaques showed significant inter-group variability in their application of bottle-manipulation strategies. This could be attributed, we believe, at least in part, to the subtle variation in local anthropogenic activities, which our methods may not have grasped in their entirety.

We found a significant positive correlation between the number of bottle-directed sophisticated or non-sophisticated manipulative techniques, performed by individual members of the DV group within each event of fluid extraction and the time required to solve each such Task-II event. Certain individual macaques, who were proficient in bottle manipulation were thus able to access the liquid contents of the bottle quickly and efficiently. Moreover, our preliminary analysis of these events and the individuals performing them showed that, as subjects gained more experience with bottles, they performed fewer and more effective manipulative techniques on them. We also observed that the transition probability of switching from comparatively sophisticated to non-sophisticated manipulative techniques increased during longer liquid-extraction events (Pal et al., pers. obs.). In other words, the subject macaques introduced non-sophisticated techniques more frequently when they were initially unsuccessful in extracting the liquid from the bottles although they employed more sophisticated techniques. If, for example, individuals were unable to unscrew a bottle with a sophisticated technique, due to its cap getting stuck between two screw threads, they rapidly resorted to the use of unsophisticated, but often more efficient, manipulative techniques, such as biting and perforating the bottles. Our study thus suggests that the observed intra-and inter-individual bottle-directed behavioral variability, which we refer to as behavioral flexibility (see Lea et al., 2020 and references therein) in the study population could be largely context-driven. Therefore, even though the study macaques may have employed manipulative techniques, some sophisticated, which could have been shaped and refined by social and individual learning, certain older techniques, belonging to previously expressed schemata, could conceivably re-emerge under certain conditions during particular bottle-manipulation events.

Contrary to the female-biased success in bottle manipulation, reported by Mangalam and Singh (2013) less than a decade ago, we found no significant effect of the sex of the actor on the overall outcome of this object-directed behavior, regardless of whether it involved the use of sophisticated techniques or not. It is noteworthy that bottle-directed extractive foraging behavior was a rather recent innovation in this population, at the time of the earlier study, and limited to a few members of only one group. We believe that, during the onset and the initial social diffusion of this novel behavior in a female-bonded society, presented by the bonnet macaques, strong and stable social relationships amongst the females may have caused a female-biased acquisition of bottle manipulation within the group. In our study, the similar success in bottle manipulation across both males and females suggests that, when inhabiting such natural resource-depleted, anthropogenic environments, where human-origin foods become the major components of the macaques’ diet, all individuals, irrespective of sex, would be motivated to search for these alternative food sources.

There was, nevertheless, a male-bias in the use of sophisticated bottle-opening techniques, which we recorded in our study population. This was possibly due to the PL group, in which, interestingly, none of the females, but all the adult males, performed these techniques. This sex-biased pattern is rather surprising, as all the sampled adult females in the DV group, whose home range overlapped with that of the PL group, performed sophisticated bottle-opening techniques. Moreover, we monitored four of the seven adult females, but a lesser proportion – two out of four – of the adult males of the PL group during Task I and yet, did not observe the females perform any of these techniques. We, therefore, wonder whether there is some difference in the capabilities of male and female bonnet macaques to acquire proficiency in the performance of sophisticated, extractive foraging techniques.

We, however, wonder whether the prevailing dominance hierarchy in particular macaque groups, as well as the temperamental profiles of the individual macaques themselves, could lead to the more socially dominant males of the group displacing other individuals, both female or male, but below them in the social hierarchy, in order to monopolize the handling of bottles, independently of who originally found these bottles. The existence of such behavioral interactions within groups and/or sex-biased learning differentials will perhaps only be revealed by detailed observations on the developmental dynamics of such bottle-directed techniques across macaque groups, both within and across populations in the long term.

In addition to providing favorable conditions for the emergence of novel behavioral techniques in nonhuman individuals and populations, anthropogenic environments may also induce changes in individual dispositions and temperaments (Lowry et al., 2013; Barrett et al., 2019). To survive in human-altered landscapes, while exploring the environment in search of alternative food sources, individual nonhuman primates regularly interact with novel anthropogenic artefacts and resources, which reduce their neophobia (Forss et al., 2017), allow for the development of adaptive behavioral strategies (Ram et al., 2003; Sinha et al., 2005), and encourage risk-prone anthropophilic interactions (Brotcorne et al., 2017; Deshpande et al., 2018). Conforming to such observations, our study macaques, which have lived in close proximity with humans, in their highly anthropogenic environment for over six decades now, exhibited a strong propensity to forage and feed on human-associated food sources.

Paradoxically, of course, such behavioral modifications that enable nonhuman primates, including our bonnet macaques, to survive in anthropogenically impacted environments also build the foundations for largely negative human–alloprimate interactions and relationships, often referred to as conflict (Barrett et al., 2019). In these human–alloprimate interfaces, human attitudes and behaviors towards macaques, as well as the latter’s strong predilection for human-origin foods, in turn, predispose and influence the macaques to adopt certain behavioral attitudes and strategies – often aggressive and confrontational – towards their human adversaries (Jones-Engel et al., 2011; Priston and McLennan, 2013). While it was obvious to us that the presence of tourists, eating and drinking close to the macaques or discarding leftover foods and partially filled bottles in open garbage bins, significantly increased the probability of competitive, usually negative, interactions between the Chamundi population of bonnet macaques and human tourists (see also Fuentes and Gamerl, 2005; Maréchal et al., 2016), such resource provisioning also possibly fostered the emergence and spread of bottle manipulation in this population of macaques.

It is now well established that the recent, drastic increase in human presence, their artefacts and their activities in once-pristine macaque habitats has not only enhanced the negative behavioral responses of different macaque species to their human ‘benefactors’, as outlined above for bonnet macaques, but also severely disrupted the structure and dynamics of their social organizations and led to the development of unusual short-and long-term life-history strategies, which are often maladaptive, ultimately threatening the very survival of these populations (Sinha et al., 2005; Sinha and Mukhopadhyay, 2013; Dhawale et al., 2020). We thus propose to urgently implement specific management practices to reduce such anthropogenic impacts on our local bonnet macaque populations and foster their sustainable coexistence with humans well into the future. These would typically include a strict ban on provisioning, avoiding eating and drinking close to the macaques, establishment of macaque-resistant garbage bins and the education of tourists and local people about the detrimental, human-induced, socioecological and behavioral changes in the lives of the macaques, which are seriously endangering their very existence (Singh and Rao, 2004; Singh et al., 2011). We recognize, sadly of course, that such actions will ultimately lead to the cessation of the innovative opening of bottles by the Chamundi bonnet macaques and perhaps, to the loss of several other unique behavioral traditions that they may have established in recent times (*cf.* Sinha, 2005). We must, nevertheless, in the least, strive to ensure the survival of these last, threatened populations of this unique, ‘common performing monkey of southern India’, the ‘monkey in the town’s commons’ (Roonwal and Mohnot, 1977; Sinha, 2001).

## Data availability statement

The original contributions presented in the study are included in the article/Supplementary material, further inquiries can be directed to the corresponding author.

## Ethics statement

The animal study was reviewed and approved by the Research Ethics Committee of the National Institute of Advanced Studies, Bangalore, India.

## Author contributions

AP and AS conceptualized the study and designed the methodology with feedback from J-BL. AP and SM conducted the fieldwork and analyzed the data. AP wrote the paper with extensive input from AS and J-BL. All authors read, edited and approved the final version for submission.

## Funding

This study was a part of a project, funded by The Leakey Foundation, San Francisco, United States. AP acknowledges the support of a National Post-Doctoral Fellowship/NPDF of the Science and Engineering Research Board, Department of Science and Technology, Government of India (PDF/2020/001389). J-BL was funded by a Discovery Grant from the Natural Sciences and Engineering Research Council of Canada (2015-06034).

## Conflict of interest

The authors declare that the research was conducted in the absence of any commercial or financial relationships that could be construed as a potential conflict of interest.

## Publisher’s note

All claims expressed in this article are solely those of the authors and do not necessarily represent those of their affiliated organizations, or those of the publisher, the editors and the reviewers. Any product that may be evaluated in this article, or claim that may be made by its manufacturer, is not guaranteed or endorsed by the publisher.
